# The diagnostic and prognostic value of miR-92a in gastric cancer: A systematic review and meta-analysis

**DOI:** 10.1515/med-2021-0347

**Published:** 2021-09-16

**Authors:** Hanxu Guo, Yuhang Wang, Zhicheng Wang, Zishu Wang, Sheng Xue

**Affiliations:** Department of Medical Oncology, First Affiliated Hospital of Bengbu Medical College, Bengbu, Anhui 233000, People’s Republic of China; Department of Urology, First Affiliated Hospital of Bengbu Medical College, Bengbu, Anhui 233000, People’s Republic of China

**Keywords:** gastric cancer, miR-92a, diagnosis, prognosis, biomarker

## Abstract

**Background:**

miR-92a is believed to have a significant role in the diagnosis and prognosis of different types of tumors, but the potential impact of its expression is still controversial due to the sample size. We conducted the meta-analysis to figure out whether miR-92a could be used as a detecting tool for assessing the prognosis of gastric cancer.

**Method:**

A literature search was conducted by retrieving the Web of Science, PubMed, EMBASE, Chinese National Knowledge Infrastructure, VIP (Technology of Chongqing databases), and Wanfang databases (last updated by February 2020). The sensitivity (SEN), specificity (SPE), positive and negative likelihood ratios (PLR and NLR), diagnostic odds ratio (DOR), and area under the ROC curve (AUC) were pooled to explore the diagnostic performance of miR-92a. The pooled hazard ratios (HRs) and 95% CIs of miR-92a for overall survival (OS) were calculated to explore the prognostic performance of miR-92a.

**Results:**

Nine articles containing 11 studies were included. The pooled SEN and SPE were 0.76 and 0.79. Besides, the pooled PLR and NLR were 3.7 and 0.30, and the pooled DOR was 12. AUC was 0.84, indicating a significant value of miR-92a in gastric cancer detection. For the prognostic analysis of miR-92a in gastric cancer, the univariate and multivariate data’s poor OS were 1.37 and 2.01.

**Conclusion:**

The present meta-analysis demonstrated that miR-92a could be a potential biomarker for the detection of gastric cancer. miR-92a could also be used as a valuable indicator for predicting the prognosis of gastric cancer patients.

## 1 Introduction

Gastric cancer still represents the third common leading cause of cancer death with more than 1,000,000 cases in 2018 and an estimated 783,000 deaths (equating to 1 in every 12 deaths worldwide) [1]. Even now there are reports that conversion surgery following chemotherapy can improve survival [2]. The clinical outcome of prognosis of gastric cancer patients is still poor. Due to the advanced stage when people are diagnosed with gastric cancer, a reliable biomarker is needed to diagnose gastric cancer and to indicate the survival time of patients, especially in the early stages.

MicroRNAs (miRNAs) refer to small and non-coding, which are actually involved at the post transcriptional level and bind to the 3′-UTR of their target messenger RNA (mRNA) to inhibit expression. A large number of miRNAs have been downregulated or upregulated in human cancer and are regarded as oncomiRs or oncosuppressor miRs [3]. More and more evidence show that miRNAs are involved in many biological processes including cell proliferation, apoptosis, differentiation, invasion, and metastasis [4]. In addition, a host of miRNAs in serum/plasma have been demonstrated to be biomarkers to identify gastric cancer at an early stage [5]. miR-92a is a member of miR-17-5p and is associated with the development of several cancers, including gastric cancer. Besides, miR-92a has been reported to be an important diagnostic and prognostic tool of other cancers, such as colorectal cancer [6], non-small cell lung cancer [7], and breast cancer [8]. However, the clinical and prognostic roles of miR-92a in tumors still need to be identified more precisely. The objective of this systematic review and meta-analysis is to summarize the current knowledge regarding miR-92a and to evaluate its diagnostic and prognostic impact in patients with gastric cancer.

## 2 Materials and methods

### 2.1 Search strategy

To identify the relevant studies, we searched the databases PubMed, Web of Science, Embase, Chinese National Knowledge Infrastructure, Technology of Chongqing (VIP), and Wan Fang databases (up to 10 February 2020). In each of the databases, the keywords “miRNA-92”or “MicroRNA-92” or “miR-92” or “hsa-mir-92” or “microRNA-92” were used as search terms together with “gastric cancer” or “stomach neoplasm” or “stomach cancer” or “stomach carcinoma” or “Stomach Neoplasms (Mesh).” In addition, we also sifted through the reference lists of original articles and manually searched relevant reviews for additional literature. Before the manuscript was published, we found that there are no more articles covering this topic in other languages except English and Chinese.

### 2.2 Inclusion and exclusion criteria

To screen out eligible studies, the following specific criteria were used: (1) gastric cancer was diagnosed via histopathology; (2) the study evaluated the diagnostic or prognostic value of miR-92a in gastric cancer; and (3) sufficient data were provided to calculate the sensitivity and specificity (for diagnostic value), and to calculate the hazard ratio (HR) and corresponding 95% CI (for prognostic value).

The exclusion criteria were as follows: laboratory studies, review articles, case reports, animal studies, or studies that did not provide sufficient data to calculate the diagnostic or prognostic value of miR-92a. If the same patient population was reported in several publications, the most recent study was selected for analysis.

### 2.3 Data extraction

Two independent researchers (G.H.X. and W.Y.H.) extracted data from all the included studies. The uncertain results were assessed by another investigator (W.Y.H.). For determining the diagnostic value of miR-92a, the following data were extracted: (1) first author’s name, country, year of publication, and ethnicity of the population studied; (2) number of patients and controls; (3) assay type for evaluating miR-92a; (4) stage of gastric cancer; and (5) diagnostic results of SEN, SPE, TP, FP, FN, and TN. For the prognostic value of miR-92a, the following data were extracted: (1) first author’s name, country, year of publication, and ethnicity of the population studied; (2) number of patients and controls; (3) assay type for evaluating miR-92a; (4) stage of gastric cancer; and (5) prognostic outcomes including HRs of elevated miR-92a expression for overall survival (OS)/disease-free survival (DFS).

### 2.4 Quality assessment

For diagnostic meta-analysis, we used QUADAS-2 as a tool to assess the quality of the diagnostic value [9]. This tool includes four domains to evaluate: patient selection, index test, reference standard, and flow and timing through the study and timing of the index test and reference standard. The methodological quality graph and methodological quality summary were conducted by Review Manager (version 5.2. Copenhagen: The Nordic Cochrane Centre, the Cochrane Collaboration, 2012) (Figure 2). For prognostic meta-analysis, the quality of involved studies was evaluated with the Newcastle–Ottawa Scale (NOS) [10,11]. In addition, it can evaluate the quality of the experiment by answering eight questions. Each answer was ranged across the score from 0 to 9. Two independent researchers (G.H.X. and W.Y.H.) extracted the data and assessed whether each of the included literature met quality standards separately.

### 2.5 Statistical analysis

For diagnostic accuracy studies, the summary diagnostic index, including SEN and SPE, PLR, NLR, and DOR with the corresponding 95% CIs, were calculated. The heterogeneity between studies was determined using Cochran’s *Q* value and *I* ^2^ statistics. *I* ^2^ values <25%, 25–50%, and >50% were set to indicate mild, moderate, and significant heterogeneity. If *I* ^2^ > 50%, the random-effects model would be adopted. Otherwise, a fixed-effect model would be utilized for further analysis [12]. The summary receiver operating characteristic (SROC) curve was applied to assess the overall diagnostic accuracy, and the area under the SROC curve (AUC) were constructed for diagnostic usefulness. Subgroup analysis was carried out by dividing the studies according to the different sample size, assay type, and sample type.

For the prognostic meta-analyses, the pooled HR and its 95% CI were calculated to elucidate the link between high expression of miR-92a and corresponding OS of gastric cancer patients. Similarly, Cochran’s *Q* test and *I* ^2^ statistics were applied to evaluate the heterogeneity of the pooled results [13]. The statistical analyses were conducted by using Stata SE12.0 (StataCorp, College Station, TX, USA) and RevMan5.3 software.

## 3 Results

### 3.1 Literature search results

**Figure 1:**
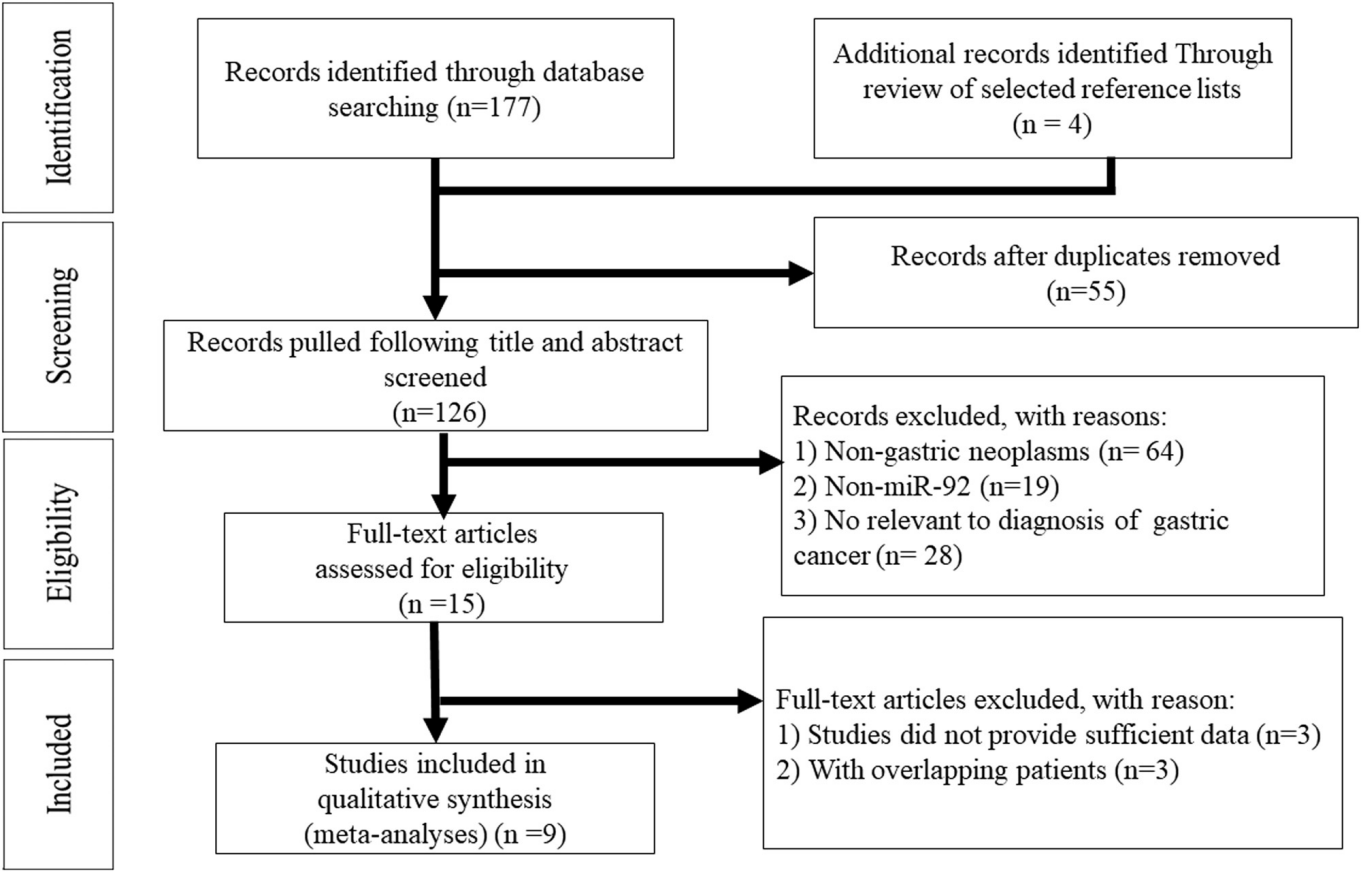
Flow chart of study selection.

The initial literature search elucidated a total of 181 articles. The identification and selection trial are briefly illustrated in Figure 1. Of course, 172 articles were excluded because they did not meet our inclusion criteria. Eventually, this meta-analysis included 9 articles covering 12 cohort studies [14,15,16,17,18,19,20,21,22].

### 3.2 Characteristics and quality assessment

**Figure 2:**
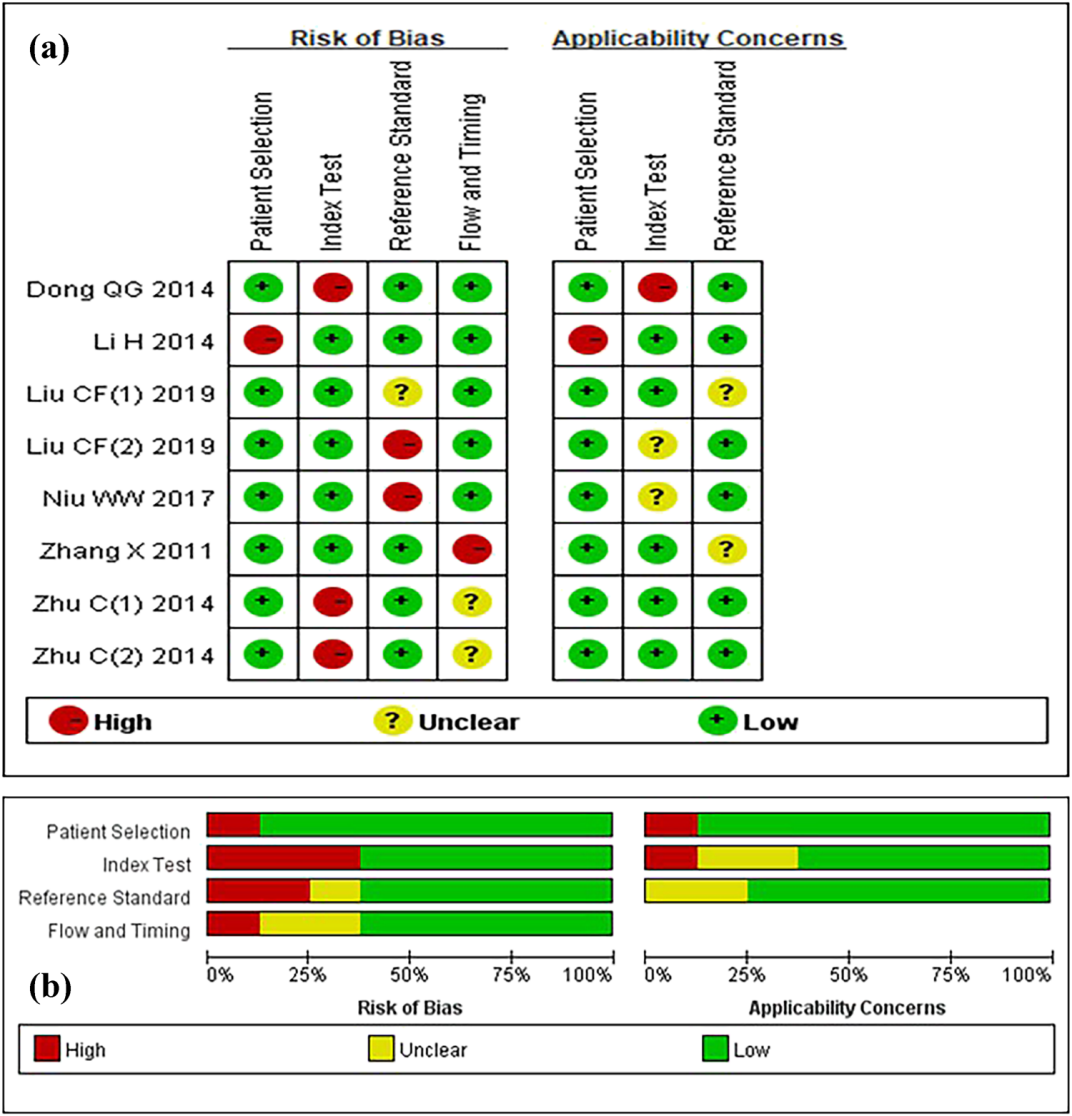
QUADAS-2 quality assessment. Investigators’ assessment regarding each domain for included studies: (a) graph and (b) summary.

For the diagnostic analysis, we included eight studies of 577 cases and 801 controls (Table 1). Besides, the studies were divided by the sample size. Gastric cancer was diagnosed using serum and plasma samples. In addition, all included studies detected miR-92a expression through quantitative Real-Time PCR (qRT-PCR) using different assay types (Taqman or SYBR). The quality of the included studies, evaluated by the QUADAS-2 assessment tool, is shown in Figure 2a and b, which is suitable for quantitative synthesis.

**Table 1:** Characteristics of the studies that related to the diagnosis of gastric cancer

Study	Country/year	Design	Sample type	Tumor/control	Stage	Cutoff	Test method	Sensitivity (%)	Specificity (%)
Zhang X	China/2011	R	Blood	80/40	I–IV	NA	RT-qPCR	85.7	70.8
Dong QG	China/2014	R	Blood	100/100	I–IV	NA	RT-qPCR	64.0	82.0
Liu CF	China/2019	R	Blood	45/89	I–II	NA	RT-qPCR	39.9	97.8
		R	Blood	125/89	III–IV	NA	RT-qPCR	39.3	84.0
Niu WW	China/2017	R	Blood	60/303	I–IV	NA	RT-qPCR	85.7	70.8
Li H	China/2014	R	Blood	79/38	I–IV	0.028	RT-qPCR	53.0	84.0
Zhu C	China/2014	R	Blood	40/40	I–IV	0.095	RT-qPCR	97.5	85.0
		R	Blood	48/102	I–IV	0.095	RT-qPCR	72.9	73.5

R, retrospective; QUADAS, quality assessment of diagnostic accuracy studies; RT-qPCR, reverse transcription-quantitative polymerase chain reaction; NA, not available.

In four of the eligible prognostic studies, 688 participants were included in the univariate analysis and 608 were included in the multivariate (Table 2). All the studies were identified for assessing for OS. NOS was used for evaluating the detailed quality of these studies (Table 3). The NOS score ranged from 0 to 8.

**Table 2:** Characteristics of the studies related to the prognosis of gastric cancer

Study	Country/year	Design	Sample type	Number	Stage	Cut-off	Test method	Outcome	HR (95%CI)
Peng W	China/2018	R	Blood	333	I–III	NA	RT-qPCR	OS	(U) 1.406 (1.041–1.898)
									(M) 1.353 (0.972–1.885)
								DFS	(U) 1.406 (0.983–2.013)
									(M) 1.309 (0.882–1.944)
Ren C	China/2015	R	Tissue	180	I–IV	NA	Microarray	OS	(U) 2.940 (2.010–4.310)
									(M) 3.340 (1.670–6.700)
Song W	China/2017	R	Blood	80	I–III	NA	RT-qPCR	OS	(U) 0.930 (0.320–2.720)

R, retrospective; QUADAS-2, quality assessment of diagnostic accuracy studies; RT-qPCR, reverse transcription-quantitative polymerase chain reaction; NA, not available; OS, overall survival; DFS, disease-free survival; HR, hazard ratio; CI, confidence interval.

**Table 3:** Newcastle–Ottawa quality assessment scale

First author	Year	Quality indicators from Newcastle–Ottawa scale								Score
		1	2	3	4	5	6	7	8	
Wu Q	2013	★	★	**★**	—	★	★	★	★	7
Ren C	2015	★	★	★	—	★	★	★	★	7
Peng W	2018	★	★	—	★	★★	★	★	★	8

1. Representativeness of the exposed cohort; 2. Selection of the nonexposed cohort; 3. Ascertainment of exposure; 4. Demonstration that outcome of interest was not present at start of study; 5. Comparability of cohorts on the basis of the design or analysis; 6. Assessment of outcome; 7. Was follow-up long enough for outcomes to occur; 8. Adequacy of follow up of cohorts.

Note: ★ and ★★ means the studies are satisfied one or two criterion below the tables.

The indicator “5” is the special one, because if there are ★★, meaning that the experiment of “Peng W” is conducted by comparability of cohorts on the basis of the design AND analysis.

## 4 Diagnosis meta-analysis

### 4.1 Diagnostic value of miR-92a in gastric cancer

**Figure 3:**
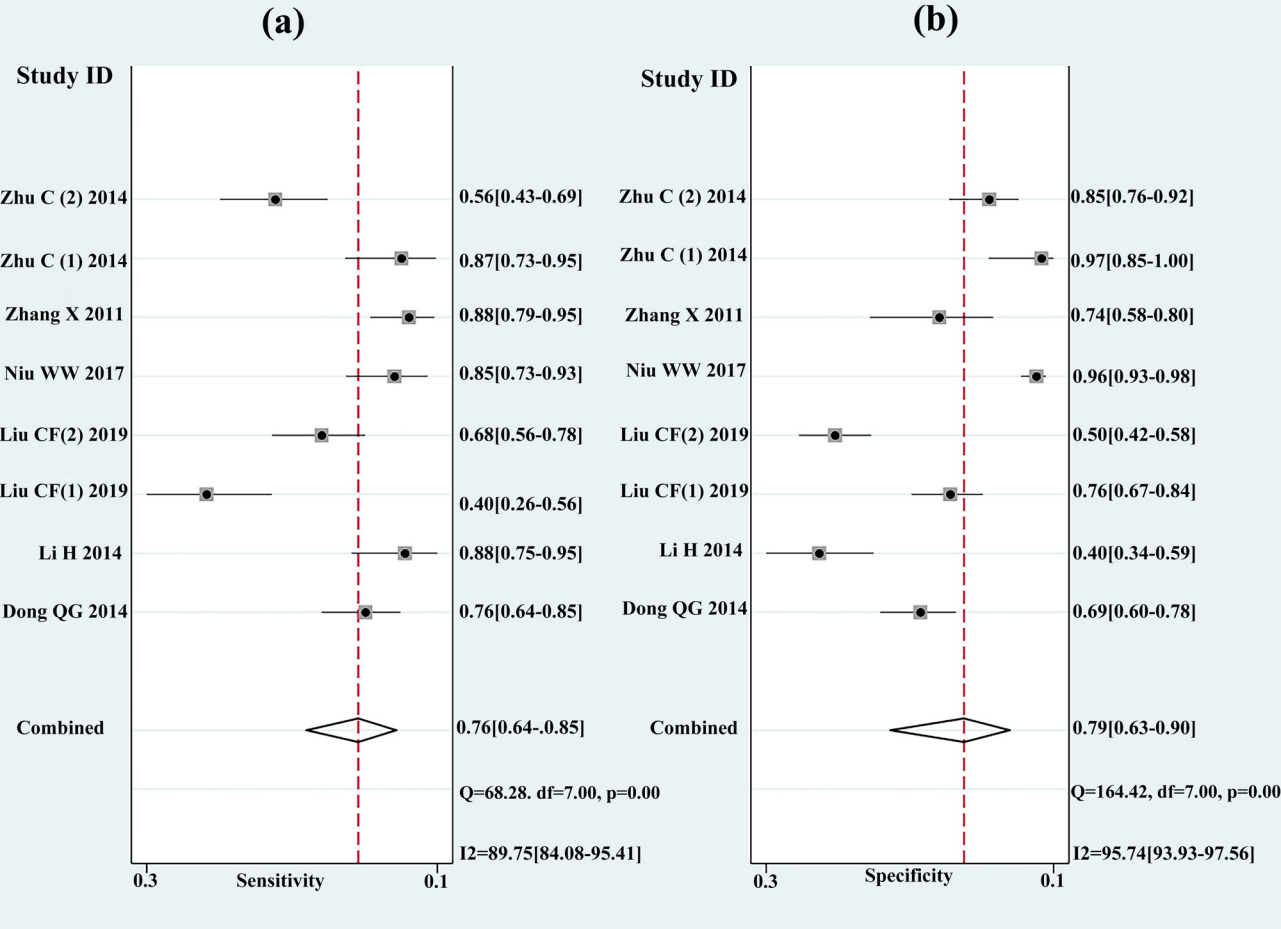
Forest plots of sensitivity (a), specificity (b) for miR-92a in the diagnosis of gastric cancer.

**Figure 4:**
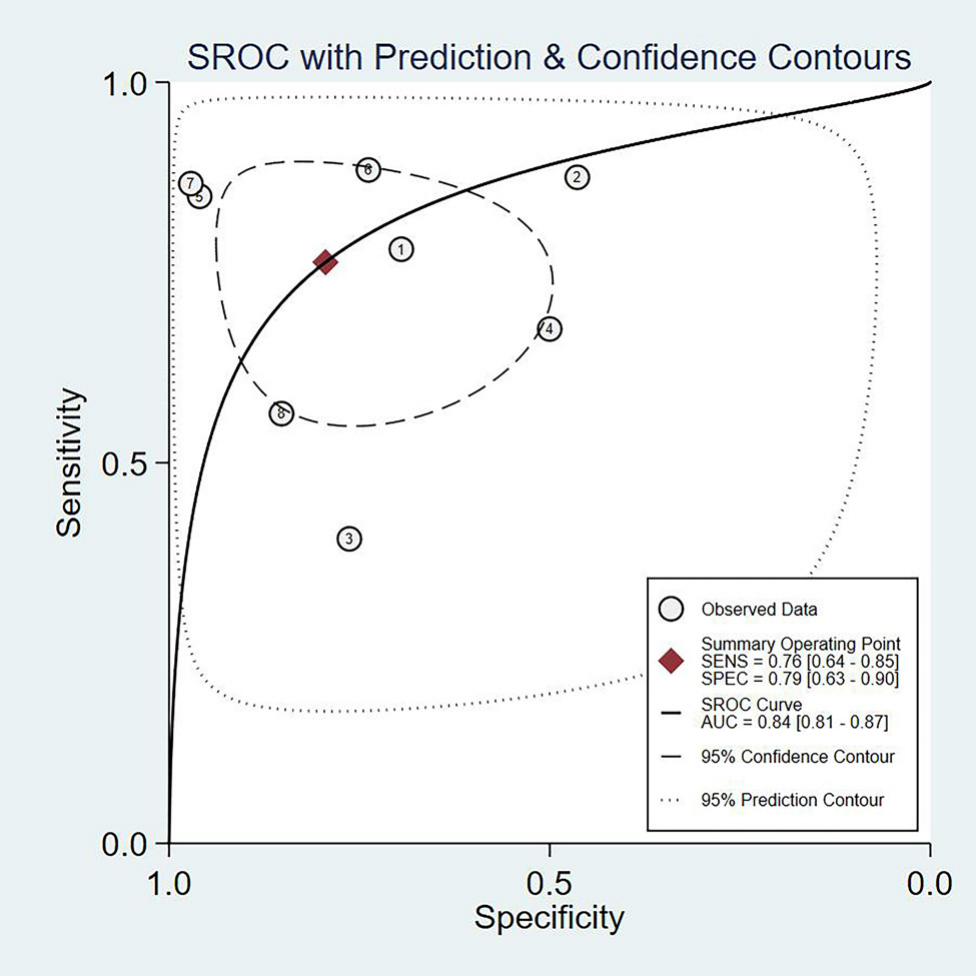
SROC curve plotted graph for the diagnostic value of miR-92a in gastric cancer.

The summary results of the diagnostic indexes for miR-92a in gastric cancer are presented in Figure 3 by using the random effect model. The pooled SEN and SPE were 0.76 (95% CI 0.64–0.85) and 0.79 (95% CI 0.63–0.90), and the pooled PLR and NLR were 3.7 (95% CI 1.8–7.5) and 0.30 (95% CI 0.18–0.50), respectively. Meanwhile, the pooled DOR was 12 (95% CI 4–38). AUC was 0.84 (95% CI: 0.81–0.87) (Figure 4). The results had significant heterogeneity (*P* < 0 01).

### 4.2 Gastric cancer

In order to analyze the heterogeneity between studies, a subgroup was performed according to the assay type, type of the sample, and sample size. As the results shown in Table 4 indicate that there are not any significant differences in the summary of sensitivity and specificity according to assay type, type of the sample, and sample size.

**Table 4:** Subgroup analysis of the diagnostic value of miR-92a in gastric cancer

	Subgroup	Sensitivity	P1	Specificity	P2
Sample size	>500	0.70 [0.54–0.86]	0.07	0.78 [0.59–0.98]	0.70
	<500	0.82 [0.70–0.94]		0.81 [0.62–0.99]	
Assay type	SYBR	0.77 [0.65–0.89]	0.79	0.73 [0.57–0.90]	0.08
	Taqman	0.74 [0.51–0.97]		0.93 [0.82–1.00]	
Sample type	Serum	0.74 [0.60–0.88]	0.24	0.73 [0.54–0.92]	0.16
	Plasma	0.80 [0.64–0.95]		0.88 [0.74–1.00]	

## 5 Prognosis meta-analysis

### 5.1 Prognostic value of miR-92a in gastric cancer

**Figure 5:**
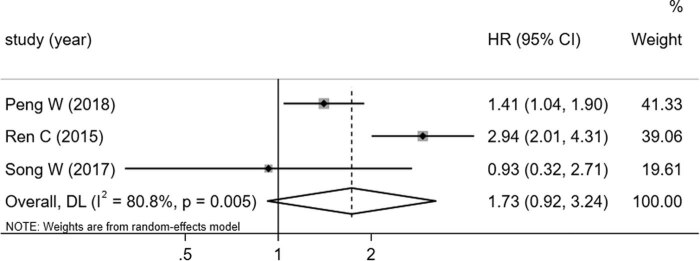
Forest plots of the studies that evaluated the HRs of high miR-92a expression on univariate study.

**Figure 6:**
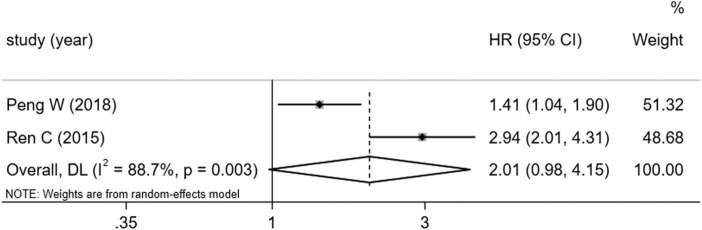
Forest plots of the studies that evaluated the HRs of high miR-155 expression on multivariate study.

The univariate (*I* ^2^ = 91.6%) and multivariate data (*I* ^2^ = 86.4%) were analyzed separately using the random effect model due to the high heterogeneity in the data. The 43 studies included for univariate analysis showed that there was no significant correction between overexpression of miRNA-92a and poor OS (HR 1.37, 95% CI: 0.92–3.24) (Figure 5). The two studies included for multivariate analysis showed that there is no significant association between the high expression of miR-92a and OS (HR 2.01, 95% CI: 0.98–4.15) (Figure 6).

### 5.2 Sensitivity analysis of the prognostic value of miR-92a expression in gastric cancer

In univariate analysis, only one study [21] used blood sample to investigate the association of miR-92a with OS. This study was omitted, and we found that the result remained similar to the overall results (HR 1.456, 95% CI: 0.620–3.416). In multivariate analysis, the study [21] in which only one used plasma was excluded found that the sensitivity result was in line with the overall results (HR 1.735, 95% CI: 0.54–5.63).

## 6 Discussion

Currently, qualifying the up- and downregulated miRNAs for the assessment of gastric precancerous lesions has also been proposed but not implemented routinely. Studies have evaluated the diagnostic and prognostic value of miRNAs in human gastric cancer with the method of meta-analysis or in systematic reviews [23]. Several miRNAs, such as miR-21 and miR-17-5p, have been proved to be potential biomarks for gastric cancers. In this study, we have evaluated the diagnostic and prognostic value of miR-92a in gastric cancer through meta-analysis. The results of the study showed that the different assay types, type of sample, and sample size did not have a significant effect on overall diagnostic accuracy. Besides, more moderate specificity and sensitivity have been found in the diagnosis of gastric cancer. We expanded the number of articles for diagnostic value compared with Wei et al.’s and Liu et al.’s studies [23,24]. In prognostic value, patients who have a high expression of miR-92a have more longer OS compared to low expression. On par with the study of Ren et al. [25], which included only two studies that investigated the prognostic value of miR-92a in gastric cancer, the present study included more articles, thus greatly enhancing the reliability of results.

The significant role of miR-92a has been found in several cancers. One study indicated that the overexpression of miR-92a is associated with osteosarcoma, colorectal cancer, and non-small cell lung cancer or hepatocellular carcinoma [26]. However, in gastric cancer, a few scholars still have definite statements on the specific role of miR-92a. The study of Liu et al. [24] of combined multi-miRNAs showed that the expression of miR-92a was increased in the serum sample of gastric cancer. Patients with high miR-92a expression have only a short survival time [26]. But one study [27] indicated that the levels of miR-92a may not be related to gastric cancer, which found contrasting results. Recently, studies by Ohzawa et al. [28] and Naruyoshi et al. [29] provided a new method for the prediction of gastric cancer, which regard the exosome miR-92a as a biomarker for the diagnosis of gastric cancer. They revealed the same results that gastric cancer patients with a high expression of miR-92a had a shorter OS time. Collectively, these conflicting results indicate the need for further studies on the role of miR-92a.

The diagnostic value of miR-92a has been demonstrated in several studies. With a sensitivity and specificity of 76 and 75%, Peng et al. [30] found that expression varied between patients with colorectal cancer and healthy controls. Moreover, their experiment indicated that the miR-92a-related combination markers achieved a higher level of diagnostic power. miR-92a also presented a high accuracy in the diagnosis of cervical cancer, with a sensitivity and specificity of 70 and 80%, respectively [31]. For gastric cancer, the diagnostic accuracy of miR-92a varied significantly, with a sensitivity ranging from 39.3 to 97.5% and a specificity ranging from 70.8 to 97.8%. In addition, there are differences among these studies, such as assay type for qRT-PCR, type of the sample, and sample size. We found that miR-92as have high accuracy in diagnosis regardless of these differences. The results indicate that miR-92a can be used as a diagnostic indicator for gastric cancer.

For the prognostic value of miR-92a, we found that the high expression of miR-92a may not be associated with poor clinical outcomes in gastric cancer patients, which had a 1.46-fold higher risk for poor OS in both univariate and multivariate studies. However, until we have finished extracting the articles, we have not found any data of DFS and progression-free survival for miR-92a in gastric cancer. Currently, non-invasive biomarkers are more and more popular for assessing survival prognosis at any time before or after treatment. In our study, we have not noticed any obvious prognostic effect on gastric cancer, which is inconsistent with the previous results of some previous prognostic studies, while our study may be the first one to report that there is a negative association between the high expression of miR-92a and patient survival. However, our sample size was larger than the previous meta-analysis. To clarify the results, more research with sufficient data is needed in future.

## 7 Limitations

This study has several limitations. (1) The sample size was still relatively small, including only 12 studies. Therefore, more well-designed studies for diagnostic and prognostic value of miR-92a are needed to obtain more reliable results. (2) The ethnicities of patients with gastric cancer varied. For example, the diagnostic meta-analysis and the prognostic meta-analysis focused only on Asians. Therefore, researchers should pay attention to the impact of the race factor in future studies. (3) We included only articles published in English and Chinese, but did not include articles in other languages. (4) Some other risk factors for the development and progression of gastric cancer need to be considered, such as Helicobacter pylori infection, unhealthy diet, etc., which will influence the reliability of the study. (5) The detection of miR-92a is based on qRT-PCR, which having used the different types of assays will affect the results of the study. Future studies should address these limitations to accurately validate the diagnostic and prognostic value of miR-92a in gastric cancer.

## 8 Conclusion

To sum up, we demonstrated for the first time that miR-92a is promising to be a novel indicator for the diagnosis of gastric cancer. And it can also be a valuable indicator for predicting the prognosis of gastric cancer. Together, these findings provide important evidence for further development of future non-invasive methods for diagnosing gastric cancer.
